# Modeling the Heterodimer Interfaces of Melatonin Receptors

**DOI:** 10.3389/fncel.2021.725296

**Published:** 2021-10-07

**Authors:** Lap Hang Tse, Yung Hou Wong

**Affiliations:** ^1^Division of Life Science and the Biotechnology Research Institute, Hong Kong University of Science and Technology, Hong Kong, SAR China; ^2^State Key Laboratory of Molecular Neuroscience, Molecular Neuroscience Center, Hong Kong University of Science and Technology, Hong Kong, SAR China; ^3^Hong Kong Center for Neurodegenerative Diseases, Hong Kong Science Park, Hong Kong, SAR China

**Keywords:** G protein-coupled receptors, dimerization, melatonin receptor, modeling, interface

## Abstract

Melatonin receptors are Class A G protein-coupled receptors (GPCRs) that regulate a plethora of physiological activities in response to the rhythmic secretion of melatonin from the pineal gland. Melatonin is a key regulator in the control of circadian rhythm and has multiple functional roles in retinal physiology, memory, immunomodulation and tumorigenesis. The two subtypes of human melatonin receptors, termed MT_1_ and MT_2_, utilize overlapping signaling pathways although biased signaling properties have been reported in some cellular systems. With the emerging concept of GPCR dimerization, melatonin receptor heterodimers have been proposed to participate in system-biased signaling. Here, we used computational approaches to map the dimerization interfaces of known heterodimers of melatonin receptors, including MT_1_/MT_2_, MT_1_/GPR50, MT_2_/GPR50, and MT_2_/5-HT_2*C*_. By homology modeling and membrane protein docking analyses, we have identified putative preferred interface interactions within the different pairs of melatonin receptor dimers and provided plausible structural explanations for some of the unique pharmacological features of specific heterodimers previously reported. A thorough understanding of the molecular basis of melatonin receptor heterodimers may enable the development of new therapeutic approaches against aliments involving these heterodimeric receptors.

## Introduction

Melatonin (*N*-acetyl-5-methoxytryptamine) is a neuroendocrine hormone which regulates multiple physiological and neuroendocrine functions. In humans, the functions of melatonin are mainly mediated by two subtypes of melatonin receptors: MT_1_ and MT_2_. Melatonin receptors are expressed in various brain regions such as the hypothalamus, hippocampus, and pineal gland, as well as in the retina and a host of peripheral tissues and organs that range from arteries, liver, and skin to the immune system [reviewed in Liu et al. (2016) and Cecon et al. (2018)]. One of the major roles of melatonin receptors is to regulate the circadian rhythm by directly acting on the suprachiasmatic nucleus of the hypothalamus (Waly and Hallworth, 2015). Melatonin receptors also have regulatory actions on sleep, immune functions, retinal physiology, and blood glucose [reviewed in Pandi-Perumal et al. (2008)].

The melatonin MT_1_ and MT_2_ receptors belong to the Class A G protein-coupled receptor (GPCR) superfamily and they share a high sequence identity of 68%. Upon activation, both receptors regulate similar signaling pathways primarily via G_*i/o*_ proteins and β-arrestin1/2 (Witt-Enderby et al., 2003), although they can also signal through G_*q/*11_ and G_16_ proteins depending on the cellular milieu (Brydon et al., 1999; Chan et al., 2002). Despite the high sequence similarity, differences in the intrinsic signaling capacity of MT_1_ and MT_2_ have been documented: MT_2_ has been shown to inhibit cGMP production (Petit et al., 1999) while MT_1_ can activate G_*s*_ (Chen et al., 2014) in some experimental models.

In the classical view, GPCR signaling is based on a ternary complex consisting of a receptor monomer, a ligand, and a G protein heterotrimer. However, a new paradigm involving receptor dimerization (and even oligomerization) has emerged as an important concept in GPCR signaling (Terrillon and Bouvier, 2004; Wang et al., 2018). The notion of GPCR dimerization/oligomerization has garnered substantial evidence from cross-linking assays, quantitative luminescence/fluorescence studies (e.g., FRET and BRET), X-ray resolved oligomeric structures, as well as computational analyses (e.g., molecular dynamics simulations). A large number of GPCR homo- and heterodimers have been documented (Prinster et al., 2005; Milligan et al., 2019). This change in the fundamental concept of GPCR signaling has brought along a huge impact on drug development. Hence, a thorough understanding on the specificity of GPCR connectivity is essential for the design of novel drugs that target receptor dimers.

Dimerization of melatonin MT_1_ and MT_2_ receptors with each other or with other GPCRs has been reported (Table 1). Co-immunoprecipitation and BRET assays in HEK 293 cells have confirmed that MT_1_ and MT_2_ can form constitutive homo- and heterodimers (Ayoub et al., 2002, 2004). A distinct signaling capacity of the MT_1_/MT_2_ heterodimer was further demonstrated in W661 cells that express both receptors endogenously (Sánchez-Bretaño et al., 2019), wherein the heterodimer mediates the melatonin-induced inhibition of adenylyl cyclase and phosphorylation of AKT/FoxO1. MT_1_/MT_2_ heterodimers are also reported to mediate the effect of melatonin on light sensitivity of mouse rod photoreceptor cells (Baba et al., 2013). In both studies, depletion of either protomer would disrupt signaling, indicating the requirement of a cooperative action of MT_1_ and MT_2_. GPR50, an orphan receptor which is structurally related to the melatonin receptors with no melatonin binding capacity, can also heterodimerize with either of the melatonin receptors in a constitutive manner (Levoye et al., 2006; Dufourny et al., 2008). The heterodimerization of GPR50 with MT_1_ abolishes the agonist binding and G protein-coupling capacity of the MT_1_ protomer, whereas it does not alter that of MT_2_ in the MT_2_/GPR50 heterodimer (Levoye et al., 2006). Another Class A GPCR, serotonin receptor 2C (5-HT_2*C*_) has been reported to form dimers with MT_2_, wherein the serotonin-induced G_*q*_ signaling pathway of 5-HT_2*C*_ is amplified upon dimerization with MT_2_ (Kamal et al., 2015). Interestingly, a melatonin-mediated unidirectional transactivation of 5-HT_2*C*_ protomer is associated with the MT_2_/5-HT_2*C*_ heterodimer (Kamal et al., 2015).

**TABLE 1:** Heterodimers of melatonin receptors.

Heterodimers	Tissues/cells	Functions/properties	References
MT_1_/MT_2_	Mouse rod photoreceptor	Mediated melatonin-induced light sensitivity rod photoreceptors	Baba et al., 2013
	661W cells	Disruption of the heterodimer abolished melatonin-induced inhibition on cAMP	Sánchez-Bretaño et al., 2019
	HEK 293 cells	Form constitutive dimers Both ligand binding sites of MT_1_ and MT_2_ preserved ligand binding capacity and selectivity Occupation of either binding site is able to induce a conformational change within the heterodimer	Ayoub et al., 2002, 2004
MT_1_/GPR50	HEK 293 cells	Abolished high-affinity agonist binding of MT_1_ Abolished G protein coupling to MT_1_	Levoye et al., 2006
MT_2_/GPR50	HEK 293 cells	No apparent change in ligand binding affinity	Levoye et al., 2006
MT_2_/5-HT_2*C*_	HEK 293 cells	Amplified the serotonin-mediated G_*q*_/PLC response Triggered melatonin-induced unidirectional transactivation of the 5-HT_2*C*_ protomer	Kamal et al., 2015

The formation of different melatonin receptor heterodimers is likely to serve as a regulatory mechanism in specific cell types to exert distinct functions. However, despite the validation of heterodimerization of melatonin receptor subtypes by proximity-based assays and co-immunoprecipitation (Cecon et al., 2018), the extent of the cooperativity across the dimer interface remains largely unexplored. GPCR heterodimerization has been extensively studied by biophysical and biochemical methods (Angers et al., 2001; Vidi et al., 2010; Borroto-Escuela et al., 2016; Fuxe and Borroto-Escuela, 2016), yet the structural basis of their interactions remains poorly understood. For a GPCR such as MT_2_ that can form distinct heterodimers, a key question is whether it dimerizes with different partners via the same interface. Here, we used computational approaches to examine the preferred interface on the MT_2_ protomer across different MT_2_ heterodimers to further understand the structural basis of dimer formation.

## Materials and Methods

### Homology Modeling

The homology models of human CXCR_4_, MOR, DOR, MT1, MT2, GPR50, and 5-HT_2*C*_ in an inactive conformation were constructed using the Modeler 9.24 (Webb and Sali, 2016). Protein sequences of the human CXCR_4_, MOR, DOR, MT_1_, MT_2_, GPR50, and 5-HT_2*C*_ were obtained from the UniProt database.^1^ Mutations in the crystal structures of CXCR_4_ (PDB code 3ODU), MOR (PDB code 4DKL), DOR (PDB code 4N6H), MT_1_ (PDB code 6ME2), MT_2_ (PDB code 6ME6), and 5-HT_2*C*_ (PDB code 6BQH) were rebuilt into wild-type structures. Intracellular loop 3 (IL3) of the receptors was omitted by chain break to avoid uncertainty during the loop prediction. The model of GPR50 was generated based on the crystal structure of its most closely related receptor, MT_1_ (51.1% identity, PDB code 6ME2). Selection of the final homology models was based on the DOPE scoring function (Shen and Sali, 2006) and visual inspection, followed by validation using PROCHECK^2^ (Laskowski et al., 1993; Rullmann, 1996).

### Membrane Protein Docking

Rosetta 3.12 MPdock was used to study the potential interfaces of melatonin receptor heterodimers (Gray et al., 2003; Chaudhury et al., 2011; Alford et al., 2015). The homology models of CXCR_4_, MOR, DOR, MT_1_, MT_2_, GPR50, and 5-HT_2*C*_ were firstly superposed on GPCR dimer structures representative of interfaces I, II, and III to obtain initial dimer structures of CXCR_4_/CXCR_4_, MOR/DOR, MT_1_/MT_2_, MT_1_/GPR50, MT_2_/GPR50, and MT_2_/5-HT_2*C*_ with each pair in three distinct orientations. The dimer templates 6OFJ (Interface I), 5O9H (Interface II), and 4DKL (Interface III) were chosen based on the inactive conformation of receptors and the relative orientation between protomers that allow more flexible docking. The membrane spanning information of the initial dimers were obtained from PPM server (Lomize et al., 2012) and span files that contain the spanning topology of proteins were generated with Rosetta 3.12 (Alford et al., 2015). A pre-packing step was undertaken in membrane embedding constant, which optimizes side chains of protomers before docking. Ten models were generated for each heterodimer pair and each interface, and the model with the lowest Rosetta total energy was selected as the input for the membrane protein–protein docking using Rosetta MPdock (Alford et al., 2015). For each heterodimer pair, 1,000 models were generated for interfaces I, II, and III. The top 50% model output with lower Rosetta total score were sorted according to their Rosetta interface score, and the best 10 models for each interface were exported for further analysis.

### Scoring, Analysis, and Mutagenesis of the Predicted Heterodimer Models

The top 10 models for each orientation of a dimer pair were then submitted to the PRODIGY web server^3^ to assess the free energy of binding and dissociation constant (at 37°C; Xue et al., 2016). The interface area of models was measured by UCSF Chimera using default parameters (Pettersen et al., 2004; Ray et al., 2005). Receptor structures visualization and *in silico* mutagenesis was performed using PyMOL Molecular Graphic System 2.1 (Schrodinger, LLC).

## Results

### Homology Models

The homology models of C–X–C chemokine receptor type 4 (CXCR_4_), μ-opioid receptor (MOR), δ-opioid receptor (DOR), MT_1_, MT_2_, GPR50, and 5-HT_2*C*_ were built by Modeler 9.24 and assessed with the PROCHECK server with the Ramachandran plots shown (Supplementary Figure 1). According to the PROCHECK evaluation, 94.4, 97.8, 96.6, 96.3, 95.1, 91.1, and 90.3% of the residues are in the most favored regions for CXCR_4_, MOR, DOR, MT_1_, MT_2_, GPR50, and 5-HT_2*C*_, respectively. No residues are found in disallowed regions for all the models. All homology models share a high structural similarity with their corresponding crystal structures (i.e., CXCR_4_ vs. 3ODU, MOR vs. 4DKL, DOR vs. 4N6H, MT_1_ vs. 6ME2, MT_2_ vs. 6ME6, GPR50 vs. 6ME2, and 5-HT_2*C*_ vs. 6BQH) with RMSD values of 0.287, 0.140, 0.141, 0.155, 0.129, 0.118, and 0.300 Å, respectively.

### Heterodimer Interfaces

**FIGURE 1:**
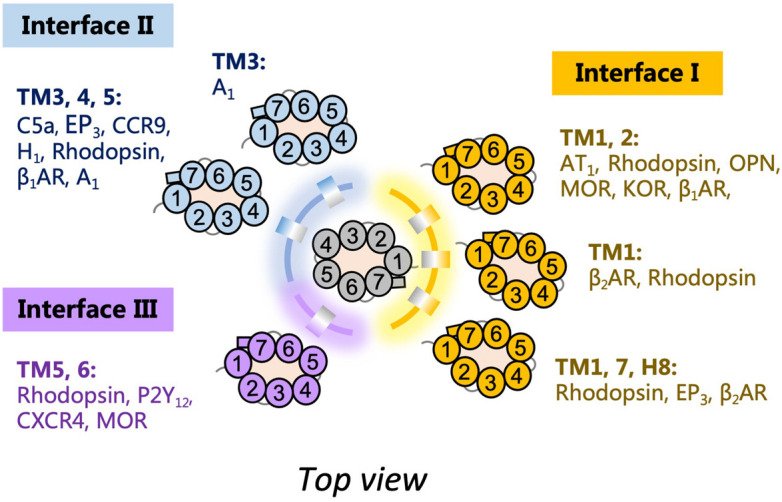
Schematic diagram of G protein-coupled receptor (GPCR) dimer interfaces I, II, and III as depicted from the top. Examples of receptor dimers bound at the different transmembrane helices (TM) regions are shown.

The assembly of GPCR dimers or oligomers can be achieved via multiple interfaces. Both symmetric [protomers interact with each other using the same set of transmembrane helices (TMs)] and asymmetric (protomers interact with each other via different sets of TMs) dimer conformations have been described, with most of the Class A GPCRs adopting the symmetric dimer interface. A list of known symmetric dimers with crystal poses is shown in Table 2. The available receptor dimer structures (Table 2) together with the previous computational studies (Kaczor et al., 2015) have unveiled three major interfaces for GPCR dimerization or oligomerization: (I) TM1, 2, 7, and/or helix 8 (H8); (II) TM3, 4, and/or 5; and (III) TM5 and 6 (Figure 1). Due to the architecture of GPCRs, it is unlikely and energy-unfavorable for dimerization to occur via the interfaces of TM2, 3 and TM6, 7 (Kaczor et al., 2015). Besides, although H8 is not in the transmembrane region, the interaction between residues on H8 can also affect the dimeric interactions and the relative orientation of protomers.

**TABLE 2:** Examples of Class A G protein-coupled receptor (GPCR) dimer structures in inactive conformation.

Interface		Receptor	Species	PDB code	References
I	TM1, H8	Rhodopsin	*Bos taurus*	6OFJ	Zhao et al., 2019
		β_2_AR	*Homo sapiens*	2RH1	Cherezov et al., 2007
	TM1, 2, H8	Rhodopsin	*Bos taurus*	2I35, 2I36	Salom et al., 2006
		OPN	*Bos taurus*	3CAP	Park et al., 2008
		MOR	*Mus musculus*	4DKL	Manglik et al., 2012
		KOR	*Homo sapiens*	4DJH	Wu et al., 2012
		β_1_AR	*Meleagris gallopavo*	4GPO	Huang et al., 2013
	TM1, 7, H8	EP_3_	*Homo sapiens*	6AK3	Morimoto et al., 2019
II	TM3	A_1_	*Homo sapiens*	5UEN	Glukhova et al., 2017
	TM3, 4, 5	C5a	*Homo sapiens*	5O9H	Robertson et al., 2018
	TM4	CCR9	*Homo sapiens*	5LWE	Oswald et al., 2016
		H_1_	*Homo sapiens*	3RZE	Shimamura et al., 2011
	TM4, 5	β_1_AR	*Meleagris gallopavo*	4GPO	Huang et al., 2013
	TM4, 5, ECL2	A_1_	*Homo sapiens*	5UEN	Glukhova et al., 2017
III	TM5	P2Y_12_	*Homo sapiens*	4NTJ	Zhang et al., 2014
	TM5, 6	CXCR_4_	*Homo sapiens*	3ODU, 3OE0, 3OE6, 3OE8, and 3OE9	Wu et al., 2010
		MOR	*Mus musculus*	4DKL	Manglik et al., 2012

**FIGURE 2:**
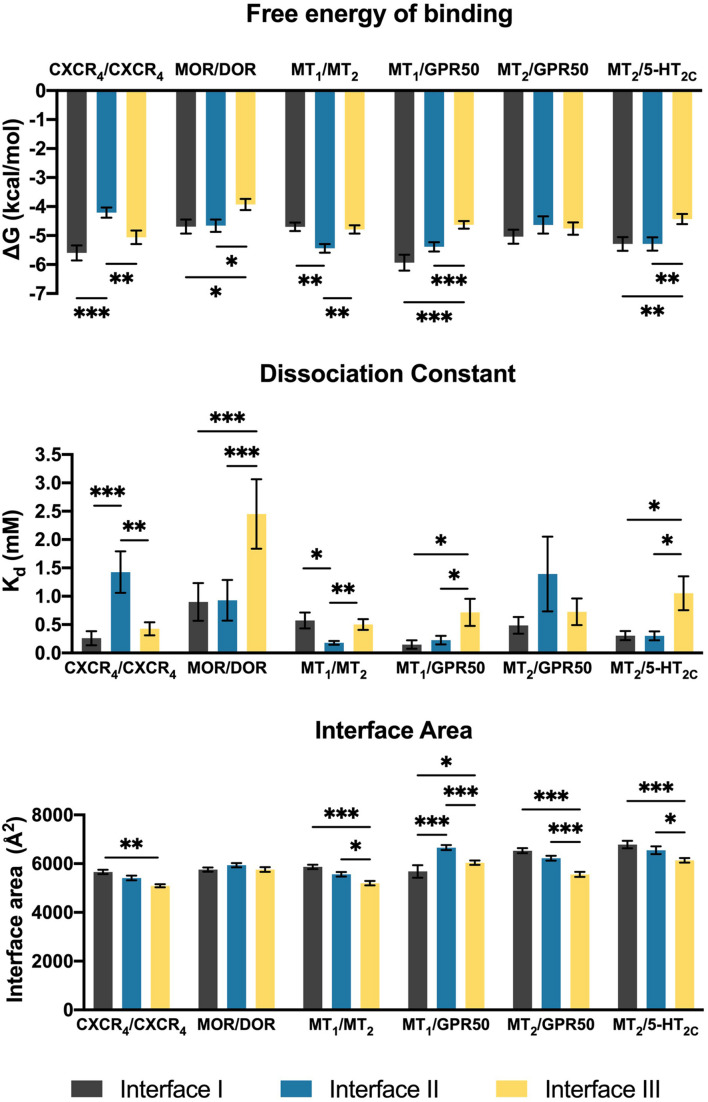
Scoring parameters calculated as average scores of top 10 models of each heterodimer with the given interface I, II, or III. Mean ± SEM is shown. Welch’s *t*-test is performed within each dimer pair; ^∗^*p* < 0.05, ^∗∗^*p* < 0.01, ^∗∗∗^*p* < 0.001.

The heterodimers of melatonin receptors (i.e., MT_1_/MT_2_, MT_1_/GPR50, MT_2_/GPR50, and MT_2_/5-HT_2*C*_) were docked into the general interfaces I, II, and III as described in the methods. For all heterodimer pairs, the best ten models for each interface were exported for protein binding energy prediction and interface area measurement (Figure 2). An interface with the lowest free energy of binding and dissociation constant would be considered as the preferred interface. In order to validate the computational pipeline, a homodimer pair CXCR_4_/CXCR_4_ with a published crystal structure was included (Wu et al., 2010). The predicted dimer models of CXCR_4_/CXCR_4_ suggested a preference of dimerization interfaces I and III (Figure 2), which recapitulated known structural knowledge and computational simulations (Wu et al., 2010; Rodríguez and Gutiérrez-de-Terán, 2012; Gahbauer et al., 2018). Another heterodimer, MOR/DOR, which was predicted by molecular dynamic simulations (Liu et al., 2009; Provasi et al., 2015) to favor dimerization via TM1, 7 and TM 4, 5 (Interfaces I and II), was also tested by our computational pipeline. Indeed, the same preference of interfaces was observed (Figure 2). Since the interface area of the predicted CXCR_4_/CXCR_4_ as well as MOR/DOR models did not show a direct relationship with the preference of dimer interfaces (Figure 2), the interface area parameter is only taken as additional supportive structural information of dimer models.

In contrast to the CXCR_4_/CXCR_4_ homodimer, interface III is generally less preferred by the melatonin receptor heterodimers (Figure 2). The MT_1_/MT_2_ heterodimer exhibited a clear preference for interface II in accordance with the parameters of free energy of binding and dissociation constant, while both interfaces I and II of MT_1_/GPR50 showed lower scores in these two parameters as compared to interface III (Figure 2). Different from MT_1_/GPR50, the MT_2_/GPR50 heterodimer did not show specific preference on any of the interfaces in terms of both parameters, while the area of interfaces I and II are significantly greater than that of interface III. As for MT_2_/5-HT_2*C*_, interfaces I and II were similarly preferred in all the scoring parameters (Figure 2). It should be noted that the dimerization between GPCRs can be constitutive or dynamic depending on the receptors. Rearrangement of the interfaces or dissociation of dimer may occur upon GPCR activation (Petersen et al., 2017; Xue et al., 2019). The dimeric models in this study are predicted as in their inactive ground states.

#### Interface II Is the Most Favored Heterodimer Interface for MT_1_/MT_2_

**FIGURE 3:**
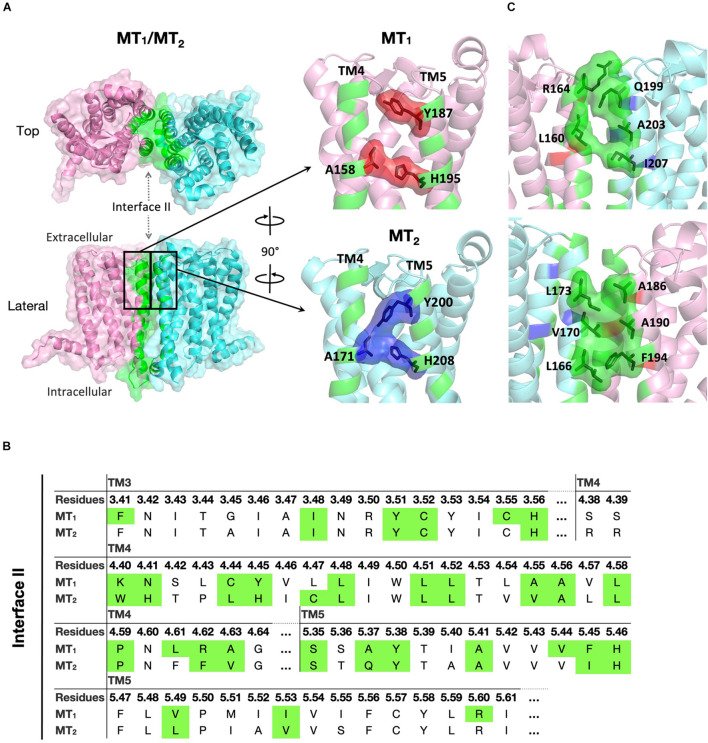
Predicted MT_1_/MT_2_ heterodimer. **(A)** MT_1_ (pink) and MT_2_ (cyan) dimerize with each other via interface II. Residues located in the interface with cutoff dASA = 1.0 are labeled in green. Residues forming the lateral ligand entrances of MT_1_ and MT_2_ are involved in the dimerization interface, and are shown in red and blue, respectively. **(B)** Predicted interface residues involved in the MT_1_/MT_2_ heterodimer. Residues involved in dimer interface II (as selected by cutoff dASA = 1.0) are labeled in green. Residues are shown in the Ballesteros–Weinstein numbering scheme (Ballesteros and Weinstein, 1995). **(C)** Side views of MT_1_/MT_2_ near the lateral ligand entrance with the orientation the same (*top*) or 180° rotated (*bottom*) as bracketed in panel **(A)**.

**FIGURE 4:**
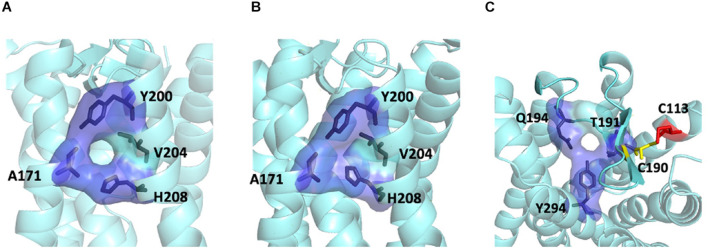
Ligand entrances of MT_2_. The lateral ligand channel of MT_2_ under the condition of crystallization (PDB code 6ME6) **(A)** or dimerization **(B)**. **(C)** The ECL opening of MT_2_ protomer in MT_1_/MT_2_ heterodimer in a top-down view, with potential disulfide bridge between C113^3.25^ (red) and C190^*ECL2*^ (yellow).

For the MT_1_/MT_2_ heterodimer, both the free energy of binding and the dissociation constant favor interface II over either interface I or III, while interface I provides the greatest interface area (Figure 2). The best model of MT_1_/MT_2_ heterodimer via interface II is depicted in Figure 3. The proposed lateral ligand entrances of MT_1_ and MT_2_ that lie between TM4 and 5 (Johansson et al., 2019; Stauch et al., 2019) and the residues forming the lateral ligand channel of both receptors [at position 4.56, 5.38, and 5.46 based on Ballesteros–Weinstein numbering scheme (Ballesteros and Weinstein, 1995)] are involved in the dimerization interface (Figures 3A,B). Therefore, dimerization via interface II is likely to hinder ligand binding. Yet, a gap was observed along L160^4.58^ and R164^4.62^ of MT_1_, and Q199^5.37^, A203^5.41^, and I207^5.45^ of MT_2_ with an estimated diameter of 2.08 Å, which is sufficiently large to enable melatonin to gain access to the lateral ligand entrances, while the other side of the melatonin receptor heterodimer is completely sealed off by A186^5.37^, A190^5.41^ of MT_1_ and L166^4.51^, V170^4.55^, and L173^4.58^ of MT_2_ (Figure 3C). The lateral ligand entrance of MT_1_ remains accessible in the lipid bilayer (Figure 3A), whereas an upward torsion of H208^5.46^ was observed in the MT_2_ protomer as compared with the reported crystal structure of MT_2_ (PDB code 6ME6), resulting in a complete seal of the lateral entrance by residues A171^4.56^, Y200^5.38^, H208^5.46^, and V204^5.42^, disallowing the ligand access (Figures 3A, 4A,B). On the other hand, an extra opening is present in MT_2_, which is formed by the open-lid conformation of ECL2 (Johansson et al., 2019) lined by residues T191^*ECL*2^, Q194^*ECL*2^, and Y294^7.39^, remains accessible for ligand entry in the MT_2_ protomer of the heterodimer. A disulfide bridge between C113^3.25^ and C190^*ECL*2^ is apparently important for holding the ECL2 in an open-lid conformation (Figure 4C). It has been shown that the ligand binding pockets of both protomers remain functional in the heterodimeric state (Ayoub et al., 2004). Our predicted model suggests that the ligand would access the binding pocket of MT_1_ via the lateral opening between protomers whilst ligand binding to MT_2_ would occur through the ECL entrance which is more accessible.

#### Differential Interface Preference by MT_1_/GPR50 and MT_2_/GPR50

**FIGURE 5:**
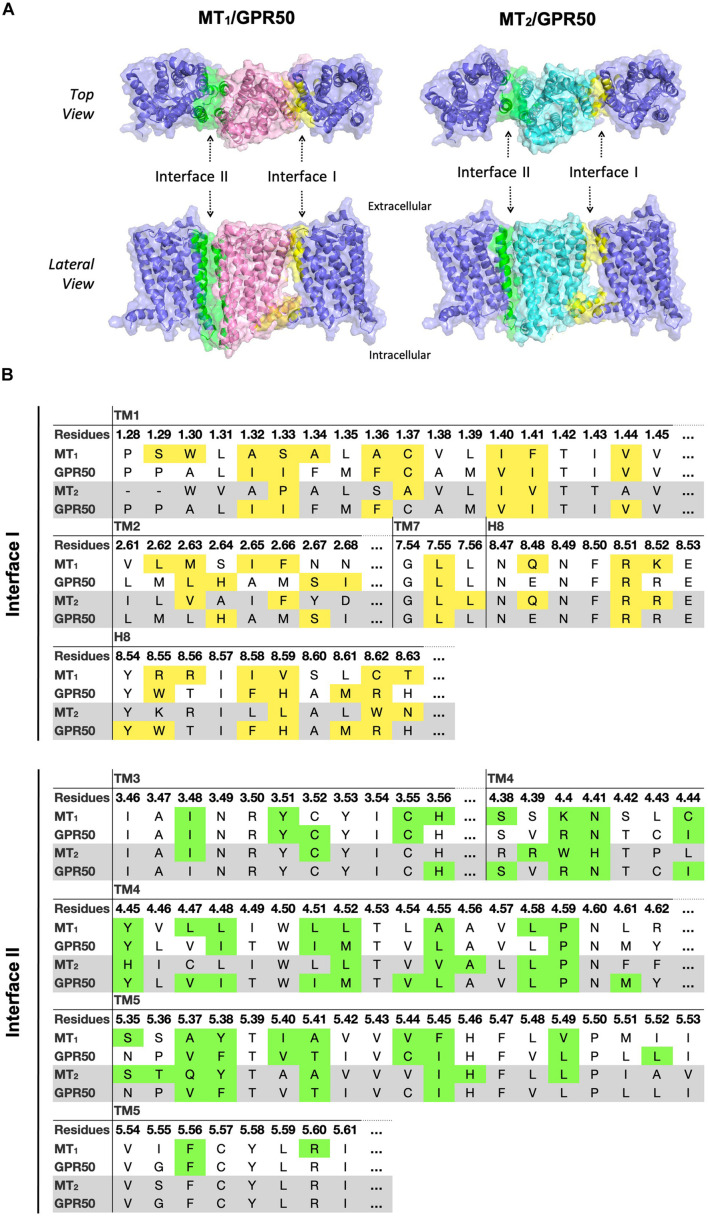
Predicted heterodimer models of MT_1_/GPR50 and MT_2_/GPR50. **(A)** MT_1_ (pink), MT_2_ (cyan), and GPR50 (slate blue) form heterodimers via interface I and II. Residues located in interface I and II with cutoff dASA = 1.0 are labeled in yellow and green, respectively. **(B)** Detailed interface residues of MT_1_/GPR50 (*white background*) and MT_2_/GPR50 (*gray background*) in dimer interface I and interface II are highlighted in yellow and green, respectively.

Although MT_1_ and MT_2_ share high structural similarity, our results suggested that MT_1_/GPR50 and MT_2_/GPR50 heterodimers have distinct preferences for dimer interfaces, which may explain the previous finding that GPR50 specifically decreases ^125^I-melatonin binding to MT_1_ but not MT_2_ (Levoye et al., 2006). The heterodimer MT_1_/GPR50 has lower free energy of binding and dissociation constant for interfaces I and II, while a larger interface area is formed at interface II (Figure 2). On the other hand, no specific preferential interface was found in the heterodimer MT_2_/GPR50 in terms of free energy and dissociation constant, while the greatest interface area formed by the MT_2_/GPR50 heterodimer is at interface I, and the smallest at interface III (Figure 2). Moreover, the dissociation constants of interface I and II of MT_2_/GPR50 are generally greater than that of MT_1_/GPR50 (Interface I: *P*-value of 0.0308 and interface II: *P*-value of 0.0042 by Welch’s *t*-test), revealing a potentially less stable condition for the MT_2_/GPR50 interhelical heterodimerization. The interface residues that lie in the transmembrane helical regions were further extracted from the top models of MT_1_/GPR50 and MT_2_/GPR50 in interfaces I and II (Figure 5). The models were selected based on the scoring parameters of free energy of binding and dissociation constant. Fewer residues are involved in the dimerization interfaces of MT_2_/GPR50 than that of MT_1_/GPR50: a total of 42 and 48 residues are involved in the dimerization of MT_1_/GPR50 heterodimer in interface I and II, respectively, as compared to 30 and 38 residues for that of the MT_2_/GPR50 heterodimer (Figure 5B).

#### Two Potential Dimerization Interfaces for the MT_2_/5-HT_2*C*_ Heterodimer

**FIGURE 6:**
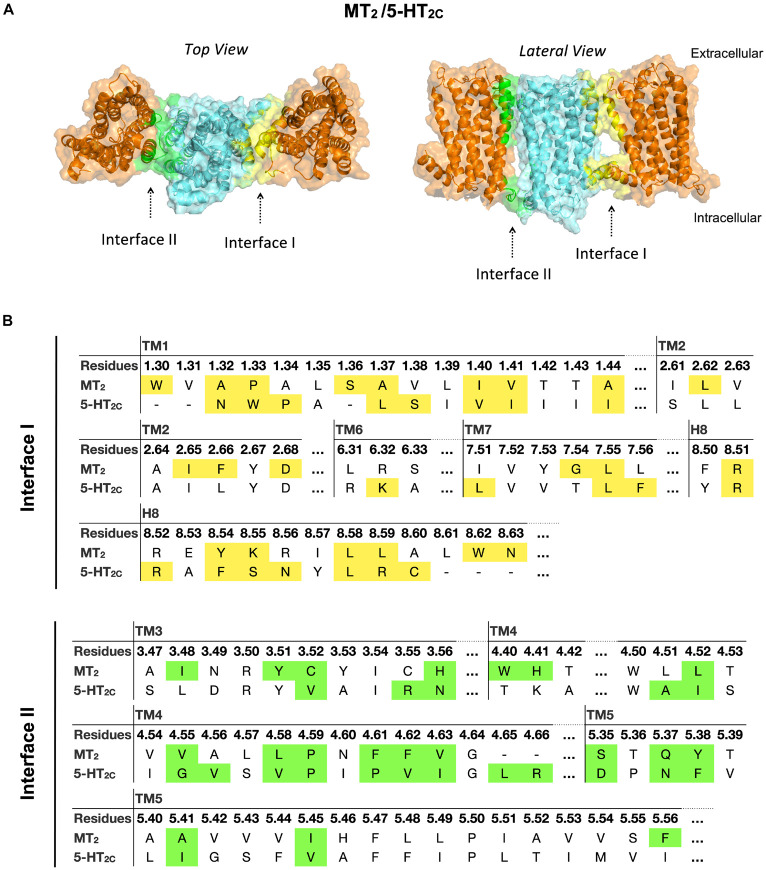
Models of MT_2_/5-HT_2C_ heterodimers. **(A)** MT_2_ (cyan) and 5-HT_2C_ (orange) are predicted to form heterodimers via interface I or II. The top view (*left*) and lateral view (*right*) of the MT_2_/5-HT_2C_ models are shown. **(B)** Predicted interface residues involved in the MT_2_/5-HT_2C_ heterodimer. Residues involved in dimer interface I and II (as selected by cutoff dASA = 1.0) are labeled in yellow and green, respectively.

In the MT_2_/5-HT_2*C*_ heterodimer, both interfaces I and II have higher scores in free energy of binding and dissociation constant than interface III, while interface I contributes the greatest surface area among all interfaces (Figure 2). The best predicted heterodimer model of MT_2_/5-HT_2*C*_ which dimerizes through interface I or II is shown in Figure 6. A previous study using the cysteine crosslinking approach has reported that 5-HT_2*C*_ can be assembled into homodimers via interface I or II (Mancia et al., 2008). Cysteine substitution of residues N54^1.32^ and W55^1.33^ successfully crosslinked the two 5-HT_2*C*_ protomers via interface I, and I192^4.63^C and N213^5.37^C mutations linked up the receptors via interface II (Mancia et al., 2008). All these residues are similarly involved in the predicted interface between MT_2_ and 5-HT_2*C*_ (Figure 6). Our results support two potential dimerization interfaces for the MT_2_/5-HT_2*C*_ heterodimer.

### Interface Residues in Melatonin Receptor Heterodimers

**FIGURE 7:**
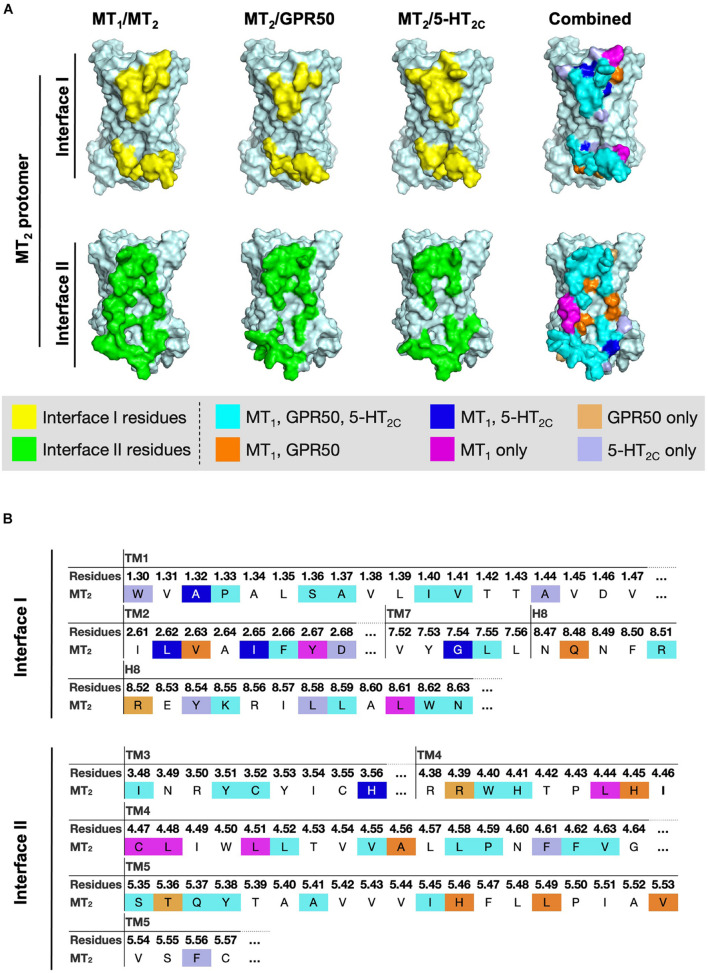
Residues of MT_2_ protomer in interface I and II of the MT_2_ heterodimer models. **(A)** The lateral interfaces I and II of the MT_2_ receptor model (pale cyan) are shown. Predicted interface residues on the MT_2_ protomer interface I or II involving in the heterodimer pairs (i.e., MT_1_/MT_2_, MT_2_/GPR50, or MT_2_/5-HT_2C_) with cutoff dASA = 1.0 are highlighted in yellow or green, respectively, and overlayed into one combined image with the indicated color codes. **(B)** The highlighted interface residues on the MT_2_ protomer in heterodimerization with MT_1_, GPR50, or 5-HT_2C_, with cutoff dASA = 1.0 are labeled with the color codes: cyan for residues involved in the interfaces of all the three heterodimers MT_1_/MT_2_, MT_2_/GPR50, and MT_2_/5-HT_2C_, orange for interface residues in both MT_1_/MT_2_ and MT_2_/GPR50, light orange for interface residues in MT_2_/GPR50 only, magenta for interface residues in MT_1_/MT_2_ only, blue for interface residues in both MT_1_/MT_2_ and MT_2_/5-HT_2C_, and light blue for interface residues in MT_2_/5-HT_2C_ only.

Since MT_2_ can dimerize with receptors not only from the melatonin receptor subfamily (i.e., MT_1_ and GPR50) but also with 5-HT_2*C*_, we further compared the interface residues used by MT_2_ protomer in the MT_1_/MT_2_, MT_2_/GPR50, and MT_2_/5-HT_2*C*_ heterodimers in interfaces I and II (Figure 7 and Table 3). The highlighted interface residues in Figure 7 are generally separated into three types: (a) interface residues commonly involved in all the three described heterodimers of MT_2_; (b) interface residues involved in two pairs of heterodimers of MT_2_ (i.e., MT_1_/MT_2_ and MT_2_/GPR50; MT_1_/MT_2_ and MT_2_/5-HT_2*C*_); (c) interface residues solely involved in the specific heterodimer pairs.

**TABLE 3:** Residues of MT_2_ involved in the predicted heterodimers.

Interface	MT_2_ residues involved in heterodimerization with:	Total number	Non-polar	Polar	Positive charge	Negative charge
I	MT_1_, GPR50, 5-HT_2C_	12	8	2	2	−
	MT_1_, GPR50	2	1	1	−	−
	MT_1_, 5-HT_2C_	4	4	−	−	−
	MT_1_ only	2	1	1	−	−
	GPR50 only	1	−	−	1	−
	5-HT_2C_ only	5	3	1	−	1
	Total^†^	26	17	5	3	1
II	MT_1_, GPR50, 5-HT_2C_	16	11	4	1	−
	MT_1_, GPR50	5	3	−	2	−
	MT_1_, 5-HT_2C_	1	−	−	1	−
	MT_1_ only	4	4	−	−	−
	GPR50 only	2	−	1	1	−
	5-HT_2C_ only	2	2	−	−	−
	Total^†^	30	20	5	5	−

*^†^Interface residues of MT_2_ involved in dimerization with any of the protomer (i.e., MT_1_, GPR50, or 5-HT_2C_).*

Because of their geometrical location, some surface residues invariably contribute to the interfaces of all three MT_2_ heterodimers examined in this study. The common interface residues range from non-polar amino acids (Ala, Val, Cys, Pro, Leu, Ile, Trp, and Phe) to polar (Ser, Try, Asn, and Gln) and charged (Arg, His) ones, with the majority being non-polar residues; only one and two positively charged residues are involved in interfaces I and II, respectively (Figure 7B, residues in cyan; Table 3).

A smaller subset of interface residues is additionally involved when MT_2_ dimerizes with distinct protomers, which may act as anchors for establishing interaction with specific protomers (Figure 7 and Table 3). Residues including Ala, Val, Leu, Gln, and His on the MT_2_ protomer are implicated in both MT_1_/MT_2_ and MT_2_/GPR50 heterodimers (Figure 7B, residues in orange). Most of these residues are found in interface II while only two residues are in interface I of MT_2_ (Table 3). Specifically shared interface residues on MT_2_ are also observed in MT_1_/MT_2_ and MT_2_/5-HT_2*C*_ heterodimers. Interface residues that are only involved in MT_1_/MT_2_ and MT_2_/5-HT_2*C*_ heterodimers in the interface I are all hydrophobic (A40^1.32^, L98^2.61^, I101^2.65^, and G309^7.54^) and may help to stabilize the inter-helical interactions, while a polar His residue (H144^3.56^) is involved in the interface II of both MT_1_/MT_2_ and MT_2_/5-HT_2*C*_ (Figure 7, residues in blue; Table 3). No overlapping interface residue is uniquely shared between MT_2_/GPR50 and MT_2_/5-HT_2*C*_ heterodimers except the commonly involved interface residues (Figure 7).

As for the interface residues solely involved in specific heterodimer pairs, the MT_2_ protomer has the fewest residues for unique interaction with GPR50 at both interfaces I and II (Figure 7B, residues in light orange). Four non-polar residues were specifically involved in the MT_1_/MT_2_ heterodimer at interface II while only two residues (one non-polar and one polar) were located on interface I (Figure 7B, residues in magenta). Conversely, interface I of MT_2_ contains more residues than interface II for specific MT_2_/5-HT_2*C*_ dimerization (Figure 7B, residues in light purple).

### *In silico* Mutations of Interface Residues on MT_2_

**FIGURE 8:**
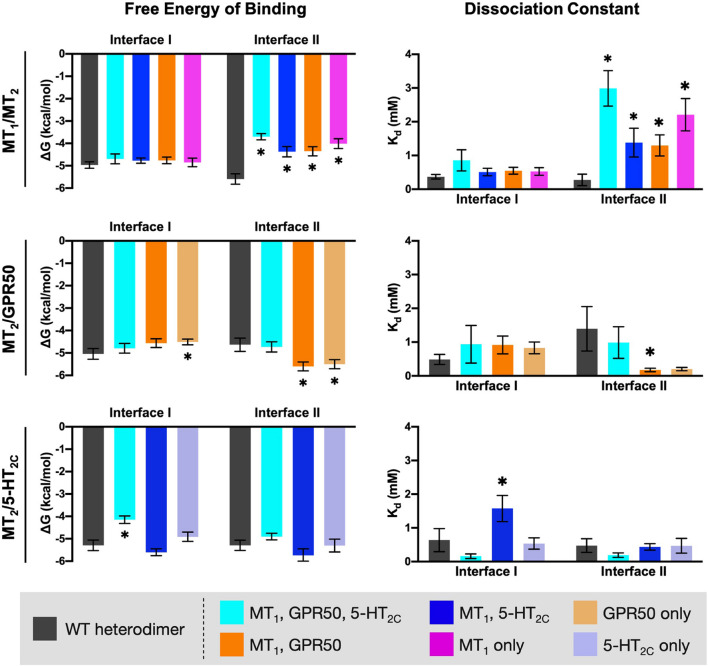
*In silico* alanine mutagenesis of MT_2_ residues putatively involved in heterodimerization. MT_2_ residues involved in the predicted interfaces of different heterodimers were mutated into Ala residues. MPDock was performed to predict the heterodimers formed by the corresponding WT protomer (MT_1_, GPR50, or 5-HT_2C_) with the WT MT_2_ (dark gray), or with the alanine mutants of MT_2_ (as depicted by color codes), at interfaces I and II. Free energy of binding (*left*) and dissociation constant (*right*) of each heterodimer were calculated as average scores of top 10 models of each heterodimer with the given interface I or II. Welch’s *t*-test is performed within each interface compared to the WT dimer pair; ^∗^*p* < 0.05.

Alanine mutagenesis is a common approach in computational studies of protein-protein interactions, as replacement of Ala residue at the interaction hot-spots can perturb the interaction and increase the free energy of binding (Massova and Kollman, 1999; Moreira et al., 2007). Each subset of the interface residues on MT_2_ (as defined in the previous section and highlighted in Figure 7) were mutated into Ala residues *in silico*, except those that are originally Ala. MPDock was performed using the wildtype (WT) or mutated MT_2_ with MT_1_, GPR50, or 5-HT_2*C*_ WTs. The best ten models for interfaces I or II were exported and the free energy of binding and dissociation constant were estimated (Figure 8). Mutating the common interface residues was expected to introduce the greatest disturbance on dimerization, as 11 out of 26 interface residues and 15 out of 30 interface residues were mutated in interfaces I and II, respectively. Indeed, for the MT_1_/MT_2_ heterodimer, mutation of any subset of interface residues (i.e., common interface residues, residues involved in both MT_1_/MT_2_ and MT_2_/5-HT_2*C*_ or MT_2_/GPR50 heterodimers, or those in MT_1_/MT_2_ only) significantly affected the scoring parameters in interface II but not interface I (Figure 8, top panels). Mutation of common interface residues in all three heterodimers (MT_1_/MT_2_, MT_2_/GPR50, and MT_2_/5-HT_2*C*_) led to the greatest perturbation in dimerization in interface II, with the free energy of binding increased from −5.59 to −3.7 kcal/mol and the dissociation constant increased from 0.276 to 2.99 mM (Figure 8, top panels). The docking result further supported that MT_1_/MT_2_ may heterodimerize via interface II. On the other hand, relatively high free energy of binding and dissociation constant were observed in the modeling of MT_2_/GPR50 heterodimer, alanine mutation of interface residues on MT_2_ did not further increase the two parameters except for the residue Arg^8.52^ (which is specifically involved in MT_2_/GPR50), which increased the free energy of binding in interface I but not the dissociation constant (Figure 8, middle panels). Interestingly, alanine mutagenesis on interface II even reduced the scoring parameters, indicating a more stable dimeric form (Figure 8, middle panels). The interaction between MT_2_ and GPR50 seems to be less dependent on the transmembrane domains. As for MT_2_/5-HT_2*C*_, the mutation of common interface residues and the interface residues shared by MT_1_/MT_2_ and MT_2_/5-HT_2*C*_ increased the free energy of binding and dissociation constant, respectively, at the interface I of the heterodimer (Figure 8, lower panels); the scoring parameters of interface II were not affected. These results suggested that all subsets of interface residues are important for MT_1_/MT_2_ dimerization, in which the mutagenesis on any group of residues can perturb the interaction between protomers. Contrastingly, *in silico* mutation of a certain subset of interface residues is insufficient to disrupt the dimerization of MT_2_/5-HT_2*C*_ in terms of free energy, the dimerization of protomers is dependent on multiple factors.

## Discussion

Melatonin, GPR50, and 5-HT_2*C*_ receptors are expressed in multiple regions of the central nervous system including the hypothalamus, cortex, and hippocampus (Giorgetti and Tecott, 2004; Hamouda et al., 2007; Gupta et al., 2017), and heterodimerization between these receptors have been reported. With the recent revelation of melatonin receptors utilizing a unique lateral channel embedded in the lipid bilayer for ligand entry (Johansson et al., 2019; Stauch et al., 2019), it becomes pertinent to establish if melatoninergic ligand binding is obstructed or unaffected in GPCR dimers containing one or more melatonin receptor protomers, since a dimer interface involving TM4 and 5 may seal off the lateral channel. The molecular modeling and computational approaches used in this study suggest that the melatonin receptors may employ interface I or II to form heterodimers, with the latter perhaps being more prevalent. Although interface II encompasses TM4 and 5, molecular modeling reveals that the lateral ligand channel remains accessible in the MT_1_/MT_2_ heterodimer. Moreover, the predicted dimerization interfaces seemingly provide a structural basis for unique pharmacological features that have been reported for the melatonin receptor and GPR50 heterodimers. It has been noted that an outward movement of TM6 is a hallmark of GPCR activation (Zhou et al., 2019). Such GPCR activation was hypothesized to impede the conformational shift of dimeric/oligomeric GPCR-G protein structures upon activation when the receptors are interacting through TM5, 6 bundles (Cordomí et al., 2015). The GPCR activation may also affect the dimer interface. A study on class C metabotropic glutamate receptors has demonstrated that the dimer interface was changed from interface II (TM4, 5) to III (TM6) upon activation (Xue et al., 2015). In this study, we are focusing on the inactive state of the melatonin receptor heterodimers and therefore the potential alteration in the activation induced- structural changes of dimers is beyond the current scope and remains to be investigated by future work.

Our computational analysis suggested a more favorable dimer condition at interface II (Figure 3) for the MT_1_/MT_2_ heterodimer, where the lateral ligand entrances of MT_1_ and MT_2_ is located (Johansson et al., 2019; Stauch et al., 2019). The ligand binding sites of both MT_1_ and MT_2_ protomers are functional in the heterodimer as revealed by radioligand binding and BRET assays (Ayoub et al., 2004), which makes dimerizing via TM4 and 5 (interface II) seems unreasonable. However, a closer look at the entrance area of the model revealed a gap between the MT_1_ and MT_2_ protomers that allows ligand access to the binding pocket of MT_1_, while the lateral entrance of MT_2_ is completely sealed. Nonetheless, the ECL ligand entrance of MT_2_ remains an open conformation maintained by the disulfide linkage between a pair of residues C113^3.25^ or C190^*ECL*2^ (Figures 3, 4), which allows molecules to pass through. In agreement with our prediction, mutagenesis study has demonstrated that alanine substitution of either residues C113^3.25^ or C190^*ECL*2^ in MT_2_ resulted in the loss of ^125^I-melatonin binding without altering the receptor surface expression (Mseeh et al., 2002), implying the importance of ECL ligand path for MT_2_. Moreover, the C113A mutant of MT_2_ remains able to heterodimerize with MT_1_ and does not prohibit the binding of ^125^I-melatonin to the MT_1_ protomer (Levoye et al., 2006). Taken together, ligand binding is likely to take place via the lateral ligand channel of the MT_1_ protomer and the extracellular opening of the MT_2_ protomer in the MT_1_/MT_2_ heterodimer.

Albeit MT_1_ and MT_2_ share high sequence identity, their dimerization with GPR50 was shown to have different functional outcomes. The ligand binding capacity of MT_1_ but not MT_2_ is diminished upon interaction with GPR50 (Levoye et al., 2006). This effect is presumably brought on by the C-terminal tail of GPR50, which impedes G_*i*_ protein recruitment by MT_1_ and hence disfavors the conformation for ligand binding (Drew et al., 1997; Levoye et al., 2006). Given that the C-terminal truncated GPR50 was able to dimerize with both MT_1_ and MT_2_, it remains unresolved as to why GPR50 specifically hinders the ligand binding of MT_1_ but not MT_2_. Our study suggested that MT_1_/GPR50 is more energy favorable to form heterodimer via interfaces I and II, while all the potential interfaces of MT_2_/GPR50 are less stable as compared to other heterodimer pairs in terms of energy (Figure 2). A relatively more stable dimeric form of MT_1_/GPR50 may accentuate the inhibition brought upon by the C-terminal tail of GPR50. Moreover, the crystal structure of MT_1_ has revealed a membrane-buried lateral ligand entrance which lies between TM4 and 5 (interface II; Stauch et al., 2019), hindrance of ligand binding of MT_1_ by GPR50 could potentially be attributed to the blockade of ligand entrance through interface II or through the allosteric conformational change induced by dimerization at the interface I. In contrast, MT_2_ has an additional extracellular ligand entry through the ECL (Johansson et al., 2019), thus blocking the lateral ligand entrance would not completely abolish its ligand binding. The structural information and our predicted interface proclivity of MT_1 *or* 2_/GPR50 heterodimers supports the notion that GPR50 does not affect ligand binding of MT_2_ while it abolishes that of MT_1_.

In the MT_2_/5-HT_2*C*_ heterodimer, dimerization via interfaces I and II is more favorable than via interface III. Indeed, the 5-HT_2*C*_ homodimer was also shown to dimerize through interfaces I and II (Mancia et al., 2008). Dimer crosslinking by I192^4.64^C and P212^5.37^C mutations can only be observed in an activated state of 5-HT_2*C*_, indicating a conformational rearrangement at the TM5 of interface II upon receptor activation, whereas crosslinking through interface I (N54^1.32^C and W55^1.33^C mutations) was observed in both active and inactive receptor states (Mancia et al., 2008). Hence, interface II is speculated to be the site for allosteric communication between receptor protomers while the role of interface I-mediated dimerization remains unclear.

The interface residues on MT_2_ involved in its heterodimer partners were highlighted and separated into different groups (Figure 7 and Table 3). As suggested by a helix packing study, amino acids with small side chains (Gly, Ala, Ser, and Cys) enable tight helix-helix packing via van der Waals interactions, and the proximity between helices further facilitates inter-helical hydrogen bonding between polar residues (Liu et al., 2004). As the commonly involved interface residues among the heterodimers of MT_2_ cover both non-polar and polar residues, this group of residues potentially represent a conserved mechanism for MT_2_ to establish receptor-receptor interactions.

It is noted that His residues in interface II of the MT_2_ protomer (H144^3.56^, H160^4.45^, and H208^5.46^) frequently participate in the interactions with specific protomers while only one His residue (H156^4.41^) is commonly involved for all the MT_2_ heterodimer pairs. In general, His amino acids putatively serve as structural determinants in specific inter-helical interactions for heterodimerization (Figure 7B). Since GPCRs are membrane proteins that also interact with lipid molecules in the membrane bilayer, residues in the TM regions may also interact with cholesterol and sphingolipids. However, the identified cholesterol binding domains [i.e., (L/V)–X_1–5_–(Y)–X_1–5_–(K/R) from N- to C-terminal direction, or (K/R)–X_1–5_–(Y/F/W)–X_1–5_–(L/V) from N- to C-terminal direction, with X can be any residue] predominantly interact with positively charged residues (Lys or Arg) instead of His, while there is no positively charged residue involved in the identified sphingomyelin-binding motif (V–X_2_–TL–X_2_–IY; Fantini and Yahi, 2015). His residues are uncommon for protein-lipid interactions, and thus the His residues in the predicted interfaces are more likely to participate in protein-protein interactions. The H144^3.56^ residue is located close to the ICL2, one of the regions known to regulate G protein coupling/activation (Flock et al., 2017), and cooperation between two protomers is observed for the G protein signals of MT_1_/MT_2_ and MT_2_/5-HT_2*C*_ (Baba et al., 2013; Kamal et al., 2015; Sánchez-Bretaño et al., 2019). Thus, H144^3.56^ may have the potential to affect G protein coupling properties upon dimerization.

Clinically relevant mutations of the GPCRs have been documented. The association between MT_2_ and T2D has been well established by experimental and genome-wide association studies (Bonnefond et al., 2012; Tuomi et al., 2016), and the identified MT_2_ variants related to T2D have been broadly categorized into three types based on their locations: at/near the ligand binding site; on the solvent-exposed intracellular side; or on the lipid-exposed intramembrane region (Bonnefond et al., 2012; Stauch et al., 2020). While the former two types of variants target the receptor functions by affecting ligand binding and downstream signaling of MT_2_, the latter type of variants may interfere with receptor dimerization or oligomerization (Stauch et al., 2020). In our predicted models, two of the T2D-associated mutants, A52^1.44^T and L166^4.51^I, lie in the heterodimer interfaces of MT_2_ with 5-HT_2*C*_ (interface I) and MT_1_ (interface II), respectively. The A52^1.44^ residue is only involved in interface I of the MT_2_/5-HT_2*C*_ heterodimer. As demonstrated by immunofluorescence staining, 5-HT_2*C*_ receptor expressed alone was mainly intracellular whilst its cell surface expression was significantly increased in the presence of MT_2_ (Kamal et al., 2015), hence the dimerization between MT_2_ and 5-HT_2*C*_ receptors may be crucial for the functioning of 5-HT_2*C*_. Besides, MT_2_/5-HT_2*C*_ displays distinct heterodimer-specific signaling with melatonin acquiring the ability to transactivate G_*q*_-coupled downstream responses (Kamal et al., 2015). Polymorphisms in the promoter region of 5-HT_2*C*_ are also associated with T2D, in which a lower promoter activity is correlated with the predisposition of obesity and T2D (Yuan et al., 2000). Therefore, the T2D associated MT_2_ variant A52^1.44^T may lead to a reduced 5-HT_2*C*_-mediated signaling or a functional impairment in the MT_2_/5-HT_2*C*_ heterodimer in the pathogenesis of T2D. This speculation is further supported by evidence that the A52^1.44^T mutation does not result in a significant functional change of MT_2_ receptor alone as compared to wild-type (including G_*i*1_ and G_*z*_ protein activation, β-arrestin 2 recruitment and ERK activation; Karamitri et al., 2018). As for the L166^4.51^I variant, the mutation can cause a defect in the melatonin-induced β-arrestin 2 recruitment while increasing the spontaneous G_*z*_ activation by MT_2_ (Karamitri et al., 2018). However, the spontaneous β-arrestin 2 recruitment and G_*i*1_ activation were similar to that of wild-type MT_2_ (Karamitri et al., 2018). One should not rule out the possibility that the L166^4.51^I mutation interferes MT_1_/MT_2_ heterodimerization at interface II. Although the association between MT_1_ and T2D has not been established, the implication of MT_1_ in T2D has been demonstrated in genetic knock-out mice, since MT_1_-deficient mice exhibit increased insulin resistance and impaired glucose metabolism (Contreras-Alcantara et al., 2010).

Apart from the T2D-associated MT_2_ variants, the A157^4.55^V variant of MT_1_ mutant with no obvious functional defect was apparently associated with non-24-h sleep-wake syndrome (Ebisawa et al., 1999; Chaste et al., 2010). The A157^4.55^ residue is located at the interface II of MT_1_/GPR50 and MT_1_/MT_2_ heterodimers. Given that both MT_1_ and MT_2_ are involved in mediating neuronal firing of the suprachiasmatic nucleus (known to control circadian rhythms) of the hypothalamus and regulate sleep (Liu et al., 1997; Gerdin et al., 2004; Gobbi and Comai, 2019), and GPR50 is also expressed in multiple regions of the hypothalamus (Regard et al., 2008; Sidibe et al., 2010), the A157^4.55^V mutation may affect the sleep-wake cycle by interrupting the formation or the function of heterodimers composed of MT_1_. A thorough understanding of the structural basis of melatonin receptor heterodimerization may bring new insights into disease pathogeneses and therapeutic designs. However, as computational study is speculative by nature, the predicted dimer structures remain to be tested experimentally. Mutagenesis studies that map the dimerization interface between protomers might further contribute to the structure-function relationship of melatonin receptor heterodimers and heterodimer-specific signaling mechanisms.

## Concluding Remarks

Our study has assessed putative dimerization interfaces and established plausible dimerization models for MT_1_/MT_2_, MT_1_/GPR50, MT_2_/GPR50, and MT_2_/5-HT_2*C*_ heterodimers. Computation models of these heterodimers have provided putative structural mechanisms/novel hypothesis on differential ligand binding properties between MT_1_/GPR50 and MT_2_/GPR50 heterodimers; distinct ligand access channels for protomers in the MT_1_/MT_2_ heterodimer (dimerize via interface II); two potential dimerization interfaces for MT_2_/5-HT_2*C*_ with interface II potentially affects the G protein coupling properties. Besides, we have identified interface residues that potentially determine the specificity of receptor-receptor interactions. Since the mapping of the dimer interfaces and residues are based on computational predictions, validation studies should be performed. Our modeling approach may facilitate the design of experimental studies (such as mutagenesis) in understanding GPCR dimers and their structure and function. Furthermore, by mutagenesis or using small molecules that disrupt the heterodimer interface, one may shift the equilibrium of receptor monomer, dimer, or even oligomer, and alter the compositions of heterodimers in cells. Manipulating the population of heterodimers in cells could also be a novel approach to study the physiological relevance of GPCR heterodimer and therapeutic development, which without directly interfering with the function of receptor but the communication between different signaling routes.

## Data Availability Statement

The original contributions presented in the study are included in the article/Supplementary Material, further inquiries can be directed to the corresponding author.

## Author Contributions

LT and YW contributed to the conception and design of the study and reviewed and edited the writing of the manuscript. Methodology and formal analysis were performed by LT with assistance from Ning Chen and Eunah Kim. LT prepared the original draft. Both authors contributed to the article and approved the submitted version.

## Conflict of Interest

The authors declare that the research was conducted in the absence of any commercial or financial relationships that could be construed as a potential conflict of interest.

## Publisher’s Note

All claims expressed in this article are solely those of the authors and do not necessarily represent those of their affiliated organizations, or those of the publisher, the editors and the reviewers. Any product that may be evaluated in this article, or claim that may be made by its manufacturer, is not guaranteed or endorsed by the publisher.

## Footnotes

1
www.uniprot.org/

2
https://www.ebi.ac.uk/thornton-srv/software/PROCHECK/

3
https://wenmr.science.uu.nl/prodigy/

## Supplementary Material

Supplementary Figure 1 Ramachandran plots for homology models of CXCR_4_, MOR, DOR, MT_1_, MT_2_, GPR50, and 5-HT_2C_. The red, brown, and yellow regions represent the favored, allowed, and “generously allowed” regions as defined by ProCheck.
