# Supplementary figures and images for: Modeling the Heterodimer Interfaces of Melatonin Receptors
Source: Front Cell Neurosci. 2021 Oct 7;15:725296. doi: 10.3389/fncel.2021.725296 (PMC8529217; doi:10.3389/fncel.2021.725296)

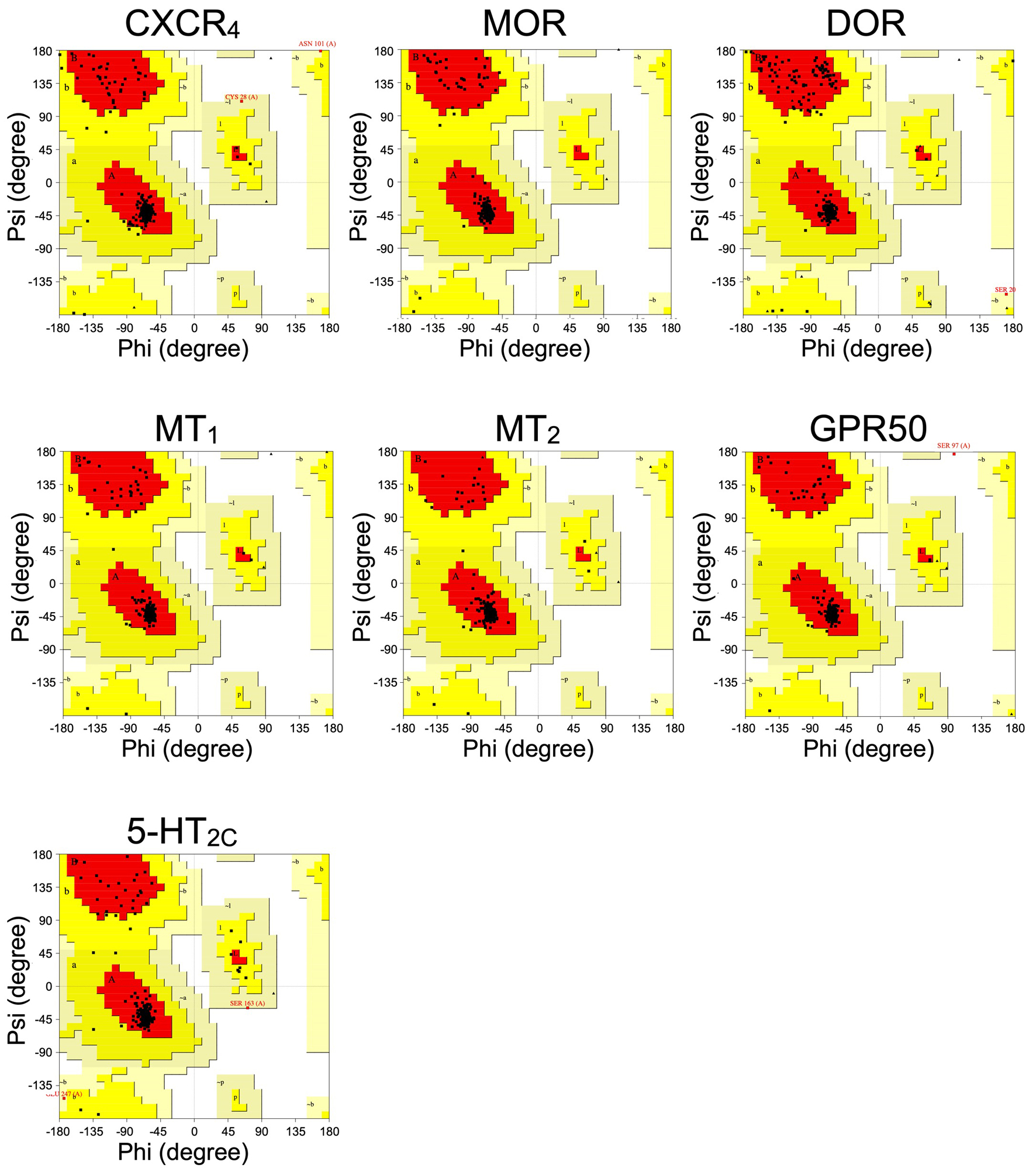

Supplement: Supplementary Figure 1 — Ramachandran plots for homology models of CXCR4, MOR, DOR, MT1, MT2, GPR50, and 5-HT2C. The red, brown, and yellow regions represent the favored, allowed, and “generously allowed” regions as defined by ProCheck. [file Image_1.JPEG]
